# The metacognitive paradox of OCD: confidence is globally reduced but shows increased sensitivity to local evidence

**DOI:** 10.21203/rs.3.rs-7609740/v1

**Published:** 2025-09-16

**Authors:** Alisa M. Loosen, Brian A. Zaboski, Avalon Moore, Calvin Bohner, Helen Pushkarskaya, Christopher Pittenger, Tobias U. Hauser

**Affiliations:** 1 Max Planck UCL Centre for Computational Psychiatry and Ageing Research; 2 Wellcome Centre for Human Neuroimaging, University College London; 3 Department of Psychiatry, Yale University School of Medicine, New Haven, CT USA; 4 Frank H. Netter MD School of Medicine at Quinnipiac University; 5 Departments of Neuroscience and Psychology, Yale Child Study Center, Yale Center for Brain and Mind Health, and Wu-Tsai Institute, Yale University, New Haven, CT USA; 6 Department of Psychiatry and Psychotherapy, Faculty of Medicine, University Tübingen, Tübingen, Germany; 7 German Center for Mental Health (DZPG), Tübingen, Germany

**Keywords:** Obsessive-Compulsive Disorder, Rule-Shifting, Decision Making, Learning, Metacognition, Confidence, Cognitive Flexibility

## Abstract

Confidence is a critical metacognitive signal that guides performance. Biases in confidence, such as excessive doubt, are hallmark features of mental health disorders, especially obsessive-compulsive disorder (OCD). Yet, the underlying neurocognitive mechanisms and how they link to learning and decision-making remain elusive. We asked patients with OCD and matched healthy controls to perform a novel rule-shifting task incorporating trial-by-trial confidence ratings. Using a Bayes-optimal model, we identified two distinct confidence biases: while patients with OCD indicated lower overall confidence, their trial-by-trial confidence ratings more accurately tracked task-relevant information, rendering their confidence reports more Bayes-optimal than those of controls. These findings challenge the idea of a simple, unified metacognitive impairment in OCD. Instead, they suggest that OCD is linked to an enhanced responsiveness to environmental evidence and feedback during decision-making.

## Introduction

Subjective confidence, our internal estimate of the probability that a choice is correct,^1^ acts as a control signal for adaptive behavior. Well-calibrated confidence determines when to exploit existing beliefs, abandon them, or seek new information, thereby supporting fast and flexible decisions in volatile environments^2–4^. In humans, low confidence increases information-seeking and strategy switches, whereas high confidence sustains ongoing courses of action^5–7^. Failures to regulate confidence can manifest clinically as chronic doubt or unfounded certainty and can hamper cognitive flexibility, the ability to update thoughts and actions as environmental demands change^8^.

Obsessive-compulsive disorder (OCD), characterized by intrusive thoughts (obsessions) and repetitive behaviors or mental acts aimed at reducing distress (compulsions),^9^ may be exemplary for how (aberrant) confidence and cognitive flexibility interact. Beyond reported deficits in executive functioning^10,11^, and cognitive flexibility^12–20^, individuals with OCD often experience chronic doubt and difficulty managing uncertainty^10,11,21–23^, alongside lowered confidence^24–30^. Notably, OCD has been characterized by a discrepancy between low confidence and objectively intact performance^27,31–34^, prompting the question of whether self-doubt in OCD reflects a difficulty in integrating external feedback or a disruption in internal self-monitoring.

To investigate these distinct possibilities, in this study, we assessed confidence judgements under changing rules in a novel task in individuals with OCD and matched healthy controls.

We find that patients with OCD achieve the same accuracy and rule-shifting performance as matched controls yet show systematically altered confidence. Using a Bayes-optimal model, we show that this mismatch between performance and confidence is driven by two distinct processes in OCD: while patients exhibit a stable, negative metacognitive bias, resulting in persistently lower confidence overall, their trial-by-trial confidence updates track external evidence more optimally than those of controls, adhering more closely to Bayesian predictions. These results indicate that persistently lowered confidence in OCD does not arise from faulty evidence integration; instead, it reflects a stable metacognitive bias that dampens subjective certainty even when environmental cues are processed. By dissociating this stable bias from dynamic evidence tracking, our findings provide a more refined understanding of the computational phenotype of OCD.

## Results

### Experimental Task and Design

**Figure 1.**
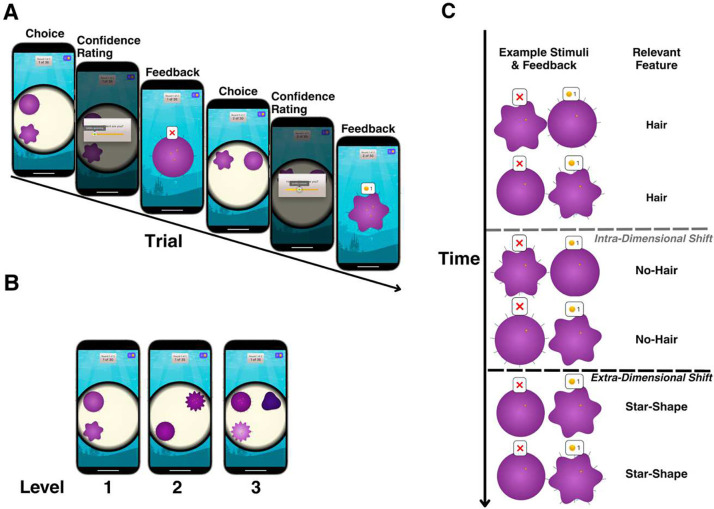
Rule-shifting task. **(A)** Participants used a touchscreen device to complete a new rule-shifting task (the “Cryptic Creatures Task”, implemented in the citizen science app Brain Explorer, www.brainexplorer.net) involving two or three multi-dimensional creatures. Each dimension (e.g., color, shape, presence of “hair”) has specific features (e.g., orange vs. blue, star vs. round, hair vs. no-hair). On each trial, participants chose one creature and then rated their decision confidence in that choice using a slider. As they moved the slider, anchor labels updated (e.g., “totally guessing,” “pretty unsure,” “pretty certain,” “totally certain”). Feedback was shown as either a coin (correct choice) or a cross (incorrect choice), with coins accumulating in the top-right corner of the screen. **(B)** Unannounced rule shifts occurred every 6–11 trials and required participants to flexibly update their decision strategy. During intra-dimensional (ID) shifts, the currently relevant dimension remained the same but switched from one feature to another (e.g., from “hair” to “no-hair”). During extra-dimensional (ED) shifts, the relevant dimension itself changed (e.g., switching from “hair vs. no-hair” to “star-shape vs. round-shape”), requiring participants to learn which new dimension was now rewarded. (C) The task progressed through three levels of increasing difficulty. At Level 1, participants were shown two creatures differing on two dimensions. At Level 2, additional dimensions were introduced (still two creatures). Finally, at Level 3, a third creature was added, making it more difficult to identify the correct dimension and feature.

We employed a novel, gamified rule-shifting paradigm, administered via a touchscreen application (cf. Figure 1A), to 29 patients with OCD and 29 healthy controls, matched for age and cognitive ability (serving as a proxy for IQ; cf. Methods). Patients with OCD, recruited from the Yale OCD Research Clinic, were clinically diagnosed, exhibited moderate-to-severe symptoms (Y-BOCS total ≥16), and were either unmedicated or on stable selective serotonin reuptake inhibitor (SSRI) treatment (cf. Methods and Supplemental Information for full participant details). On each trial of the task, participants selected one of two (levels 1–2) or three (level 3; cf. Figure 1B) multi-featured stimuli with the goal of identifying a currently rewarded stimulus feature (e.g., a particular color or shape). After making their choice, but before receiving feedback on its accuracy, they rated their confidence in their selection. Reward contingencies changed covertly and at unpredictable intervals (cf. Methods), requiring participants to continuously monitor outcomes, infer the current rule (i.e., rewarded feature), and flexibly adapt their choices and beliefs in response to environmental volatility. Changing conditions entailed both intra-dimensional [ID] shifts (e.g., from one color to another), in which the target feature shifts within the relevant dimension, and extra-dimensional [ED] shifts (e.g., from a specific color to a specific shape), in which the relevant dimension itself shifts (cf. Figure 1C and Methods). This design allowed for the trial-by-trial assessment of learning, adaptation to different types of rule shifts, and associated confidence dynamics.

### Participants Successfully Navigate the Rule-Shifting Task

To assess how well participants adapted to changing task rules across varying difficulty levels, we first assessed overall choice accuracy. A repeated-measures ANOVA on accuracy, with group (OCD vs. Controls) as a between-subject factor and difficulty level (1, 2, 3) as a within-subject factor, revealed a significant main effect of difficulty (F(2,112)=185.485,p<0.001). This indicates that accuracy decreased as difficulty increased: from level 1 $(M_{OCD}=\left.0.810,SD_{OCD}=0.049;\;M_{controls\;}=0.818,SD_{controls\;}=0.040\right)$ to level $2\left(M_{OCD}=\right.0.738,SD_{OCD}=0.063;M_{controls\;}=0.744,SD_{controls\;}=0.071$, and further at level $3\left(M_{OCD}=\right.0.573,SD_{OCD}=0.052;M_{controls\;}=0.528,SD_{controls\;}=0.078)$.

We did not observe a significant effect of group (*F*(1,56) = 0.229, *p* = 0.634) or a group × difficulty interaction (*F*(2,112) = 1.917, *p* = 0.152). We also did not find any differences when investigating average accuracy after IDs and EDs separately (cf. Supplementary Information), suggesting that the task was solved equally well by both groups.

**Figure 2.**
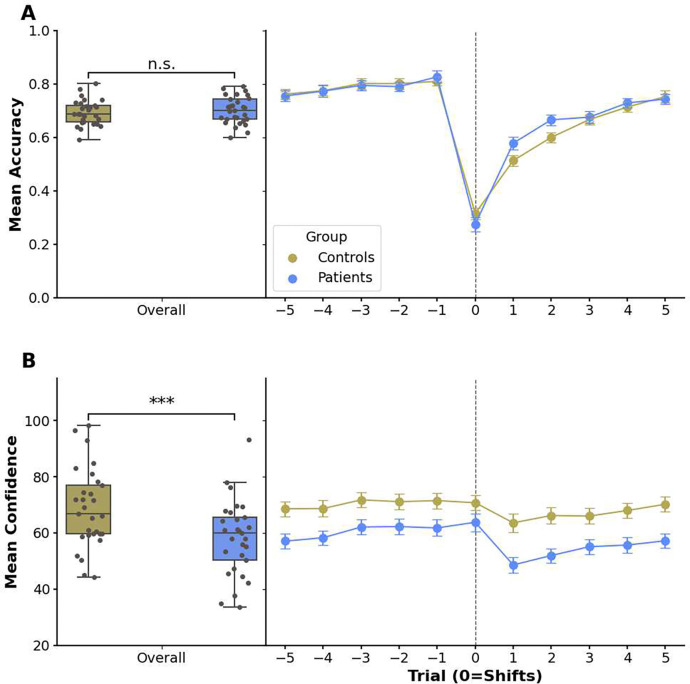
Average accuracy and confidence ratings overall and relative to rule shifts in the task. Participants played a new intra-/extra-dimensional rule-shifting task that included confidence ratings. (**A**) Participants’ average accuracy dropped when a shift happened (vertical dashed lines) but recovered afterward (right panel). Contrasting behavior between the two groups, we observed no significant difference in overall accuracy between patients with OCD and controls (left panel). (**B**) Confidence ratings dropped after the shifts and increased with more trials under the new rule (right panel). Patients were significantly less confident than controls throughout the task (left panel). The box plots on the left of each panel show the means of all trials across the entire task for each measure. The right panels display the mean behavioral measures from five trials before to five trials after each shift. Error bars represent standard errors. Statistical significance was determined using two-sided two-sample t-tests (***p<*0.001*, *n.s*. = *non-significant*).

Because such average performance metrics might obscure more subtle learning differences, we investigated whether and how quickly participants adjusted to the hidden rule shifts using logistic mixed-effects models predicting accuracy. As expected, accuracy was significantly lower on the trial following a shift (*β* = −0.912,*SE* = 0.103, *p* < 0.001). However, accuracy then recovered quickly. Pooling across groups, we examined recovery by linking accuracy to trial distance from the shift (functional form [i.e. log term] selected by Bayesian Information Criterion [BIC] from linear, quadratic, and log terms; cf. Supplemental Information). We found that accuracy increased with distance, consistent with learning the new rule (ID shifts: *β* = 1.569, *SE* = 0.079, p < 0.001; ED shifts: *β* = 0.867, *SE* = 0.068, *p* < 0.001; cf. Figure 2A right panel).

We next investigated whether the groups differed in these more fine-grained adaptation analyses and added the group variable to the mixed-effects models. Main effects for trial distance remained significant (ID shifts: *β* = 1.634, *SE* = 0.112, *p* < 0.001; ED shifts: *β* = 0.842, *SE* = 0.068, *p* < 0.001), and group main effects were non-significant (ID shifts: *β* = 0.124, *SE* = 0.172, *p* = 0.469; ED shifts: *β* = 0.111, *SE* = 0.132, *p* = 0.398). In addition, no group × distance interactions were significant (ID shifts: *β* = −0.126, *SE* = 0.155, *p* = 0.414; ED shifts: *β* = 0.024, *SE* = 0.096, *p* = 0.805), indicating comparable post-shift learning across groups.

### Participants With OCD Show Lower Baseline Confidence and Higher Feedback Sensitivity In Their Confidence Ratings

Having established comparable performance across patients and controls, we moved on to investigate potential group differences in confidence judgments. We examined average confidence ratings using the same ANOVA-structure as for accuracy (cf. Methods for details). We found a significant main effect of group (F(1,56)=8.208,p<0.01) and difficulty level (F(2,112)= 95.168, <0.001), but no significant interaction between group and task difficulty (F(2,112)= 1.386, p=0.254). Thus, while confidence declined with increased task difficulty in both groups (level 1: $M_{OCD}=68.699,SD_{OCD}=14.307;M_{controls\;}=75.244,SD_{controls}=11.588$; level 2: $M_{OCD}=62.991,SD_{OCD}=12.669;M_{controls}=72.942,SD_{controls\;}=15.719;$ level $3:M_{OCD}=41.043,SD_{OCD}=11.314;M_{controls\;}=58.170,SD_{controls\;}=16.654)$, the OCD group exhibited consistently lower confidence overall (t(56)=3.585,p<0.001; cf. Figure 2B left panel). This group difference remained significant when controlling for depression scores and was also not explained by medication status or comorbidities in the patient group (cf. Supplementary Information for additional analyses). Consistent with this pattern of lowered confidence individuals with OCD also exhibited significantly slower overall reaction times (RTs) and both groups markedly slowed as task difficulty increased (cf. Supplementary Information for RT analyses).

Next, we analyzed how confidence changed dynamically around rule shifts and in response to feedback. A significant main effect of group mirrored the lowered confidence in participants with OCD seen in the previous analysis (*β* = −9.194, *SE* = 3.032, *p* < 0.01). Moreover, confidence dropped significantly on trials following a rule shift across both groups (*β* = −5.440, *SE* = 1.656, *p* < 0.01), reflecting their uncertainty about the correct choice. Interestingly, this immediate post-shift drop in confidence was more pronounced in the patient group, as indicated by a significant interaction effect between the group variable and the variable denoting a shift on the preceding trial predicting confidence (*β* = −5.071, *SE* = 2.340, *p* < 0.05; cf. trial 1 in Figure 2B right panel).

Subsequently, confidence increased with trial distance (ID shifts: *β* = 1.236, *SE* = 0.153, *p* < 0.001; ED shifts first-order term: *β* = 258.375, *SE* = 43.591, *p* < 0.001; ED shifts second-order term: *β* = −174.328, *SE* = 38.689, *p* < 0.001; model comparison favored a linear form for ID shifts and a quadratic form for ED shifts using orthogonal polynomials, cf. Supplemental Information; cf. Figure 2B right panel). Adding the group variable yielded no significant group × trial distance interactions (ID shifts: *β* = 0.206, *SE* = 0.308, *p* = 0.506; ED shifts first-order term: *β*= 114.966, *SE*=86.367, *p*=0.188; *ED* shifts second-order term: *β*=−122.853, *SE*=76.505, *p*=0.114). Distance effects remained significant (ID shifts: *β*=1.132, *SE*=0.218, *p* < 0.001; ED shifts first-order term: *β* = 204.315, *SE*=60.750, *p* < 0.01; ED shifts second-order term: *β* = −119.424, *SE* = 52.598, *p* < 0.05) while significant group main effects again indicated significantly lower overall confidence in individuals with OCD (ID shifts: *β* = −8.611, *SE* = 3.459, *p* < 0.05; ED shifts: *β* = −11.298, *SE* = 3.313, *p* < 0.01). These findings hint towards patients having a heightened sensitivity to environmental changes (i.e. rule shifts) in their confidence ratings but ultimately adapting quickly as the new rule becomes more familiar.

**Figure 3.**
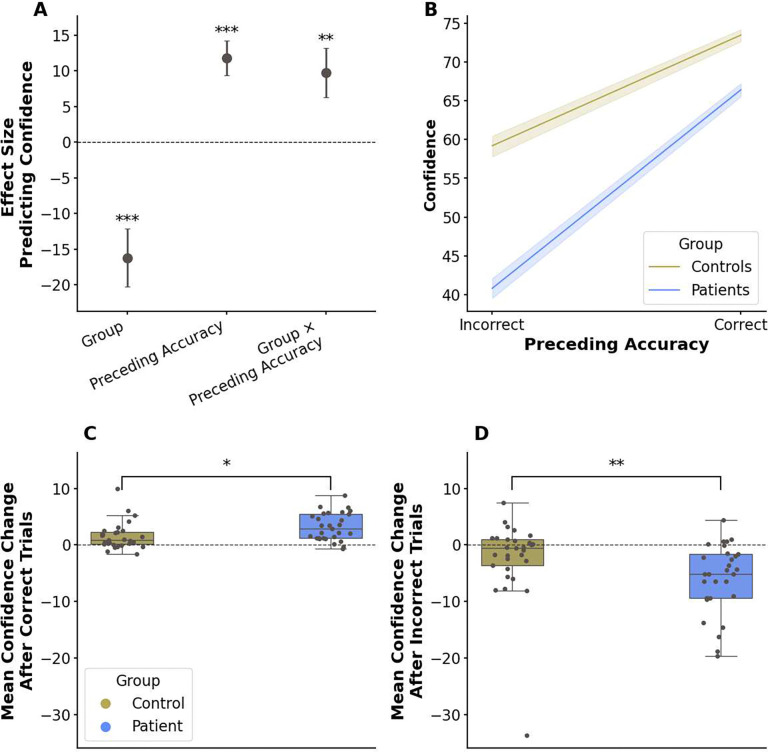
Confidence ratings in patients with OCD are more sensitive to external feedback. (**A**) A mixed-effects model indicated that, overall, patients with OCD were less confident than controls and that confidence was positively associated with accuracy on the preceding trial. (**A-B**) There was a significant interaction between group and preceding accuracy, showing that the influence of feedback on next-trial confidence differed between groups. Follow-up comparisons examining this interaction revealed that participants with OCD exhibited heightened feedback sensitivity: (**C**) they showed a significantly greater average increase in confidence following positive feedback and (**D**) a significantly greater average decrease following negative feedback compared to controls (Bonferroni-corrected). Panels (A) and (B) display model estimates or effects illustrating the main effects and interaction. Box plots in (C) and (D) represent mean confidence levels or changes on relevant trials. Error bars represent standard errors and *p < 0.05, **p < 0.01, ***p < 0.001.

To understand if the confidence ratings of participants with OCD were associated with altered sensitivity to performance feedback in general, we examined the impact of accuracy on confidence on the subsequent trial. We used a mixed-effects model predicting confidence at trial t from accuracy at trial t-1, group, and their interaction. As expected, accuracy on the previous trial positively predicted subsequent confidence (*β* = 11.779, *SE* = 2.437, *p* < 0.001; cf. Figure 3A). Consistent with overall group differences, OCD status also significantly predicted lower confidence within this model (main effect of group: *β* = −16.241, *SE* = 4.065, *p* < 0.001). Crucially, we additionally found a significant interaction between group and preceding accuracy (*β* = 9.697, *SE* = 3.445, *p* < 0.01; cf. Figure 3B), indicating that the effect of preceding accuracy on confidence was more pronounced in participants with OCD.

To further examine this interaction and directly compare feedback sensitivity, we assessed the average change in confidence after correct and incorrect trials within each group. Both participants with OCD(*t*(28) = 7.093, p* < 0.001; where * indicates Bonferroni correction) and controls (Wilcoxon signed-rank test: *W* = 57,*z* = 3.67, p* < 0.001) showed a significant average increase in confidence after correct trials. However, only participants with OCD exhibited a significant average decrease in confidence after incorrect trials (*t*(28) = −5.184, p* < 0.001); the control group’s average change did not reach significance (Wilcoxon signed-rank test: *W* = 138, *z* = −1.71, p* = 0.175). Direct group comparisons further revealed that participants with OCD showed a significantly greater increase in confidence after correct decisions (Wilcoxon rank-sum test: *U* = 598.5, *z* = 2.768, *p* * < 0.05; cf. Figure 3C) and a significantly greater decrease after incorrect decisions (*U* = 239.0, Z = −2.823, *p* *< 0.01; cf. Figure 3D) than controls.

Taken together, these behavioral analyses show two distinct characteristics of confidence ratings in the OCD group within the rule-shifting task: (1) a lower overall level (metacognitive bias) compared to controls, in the context of comparable accuracy, and (2) heightened reactivity to immediate task events like rule-shifts and performance feedback (dynamic effects). While these analyses characterize observable patterns in confidence reporting, they do not clarify whether the heightened dynamic reactivity is adaptive or maladaptive in terms of optimal evidence integration.

### Confidence Ratings of Participants with OCD are More Bayes Optimal

**Figure 4.**
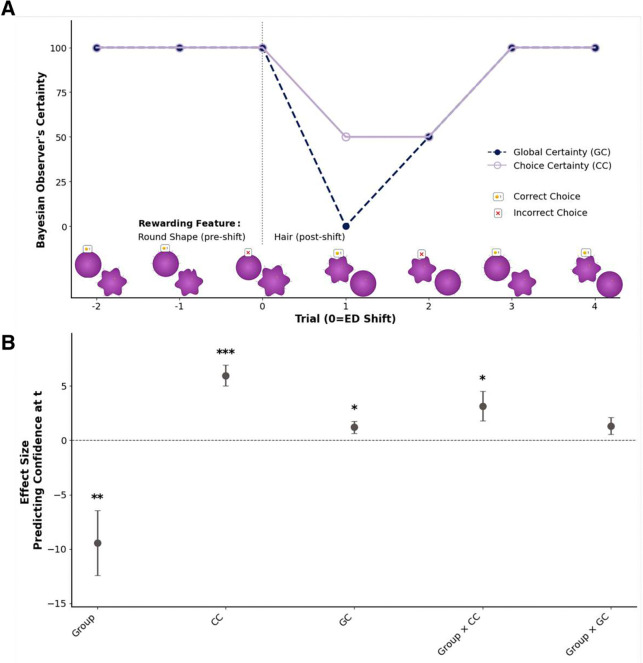
Bayesian-observer certainty is linked to confidence fluctuations. (**A**) Time course of the Bayesian observer’s Global Certainty (GC; dashed blue) and Choice Certainty (CC; purple) around an extra-dimensional (ED) rule shift, with the x-axis ranging from pre-shift trials (−2, −1), to the shift trial (extra-dimensional rule shift; dashed line at trial =0), and post-shift trials (1–4). Pre-shift, GC and CC are high under the learned “round-shape” rule. At the ED shift (0), the latent rule changes to “hair”. A negative outcome (‘X’ over the chosen stimulus) at the shift renders the previous rule incompatible, collapsing GC toward zero while CC 50, reflecting the sum of priors for the chosen features (‘hair’, ‘star-shaped’) under a belief state now divided equally between all present features (‘hair’, ‘no-hair’, ‘star-shaped’, ‘round’). On trial 1, a correct choice (yellow coin) and trial 2, an incorrect choice, allow the model to perform hypothesis elimination by reinforcing the correct features and discarding the irrelevant one. This resolution causes GC to rise, and with a final confirmatory choice on trial 3, GC and CC converge at maximal certainty, signifying that the new correct feature has been identified. (**B**) Across both groups, confidence ratings were significantly predicted by GC and CC. A significant interaction effect revealed that the relationship between CC and subjective confidence was significantly stronger in participants with OCD compared to controls, indicating more optimal tracking of choice-specific evidence in the patient group. Error bars represent standard errors and **p < 0.01, ***p < 0.001.

In this task, an optimal learner’s confidence is based not on the simple valence of feedback (positive or negative), but on the precision of the information that feedback provides. For example, an incorrect trial can lead to a subsequent increase in confidence if that feedback helps to definitively identify the correct feature. Consequently, the greater feedback reactivity observed in patients with OCD cannot be simply classified as adaptive or maladaptive. This distinction depends on the context of each trial, and the moment-by-moment accumulated evidence. To formally evaluate these confidence dynamics and disentangle stable metacognitive biases from dynamic evidence tracking, we therefore employed a (Bayes-)optimal observer model (cf. Methods). This model provides trial-by-trial estimates of optimal certainty: Bayes-optimal General Certainty (GC), reflecting overall certainty about the rewarding stimulus feature, and Bayes-optimal Choice Certainty (CC), reflecting certainty about the chosen stimulus containing the correct feature (cf. Figure 4A). While these two metrics, as expected, are positively correlated (mean within-subject: *r*_*s*_ = 0.504, *SD* = 0.125), they capture distinct aspects of optimal certainty. GC reflects the overall certainty about the current rule governing the reward contingencies (i.e., inverse entropy scaled to the confidence scale used by participants), while CC is the certainty specifically associated with the features present in the chosen stimulus, i.e. how certain the observer is that one of these features is the rewarding one given the preceding choice and reward history (cf. Figure 4A).

To relate these signals to subjective confidence, we fit linear mixed-effects models with GC, CC, group (OCD vs. control), their interactions, and covariates. We found that, in this joint model, confidence significantly tracked both normative benchmarks, with a larger effect for CC (*β* = 5.953, *SE* = 0.966, *p* < 0.001) than GC(*β* = 1.217, *SE* = 0.556, *p* < 0.05; cf. Figure 4B, Supplemental Information).

We examined how the relationship between confidence and the normative predictors differed between the OCD and control group. First, we found a persistent baseline difference: a significant main effect of group (*β* = −9.429, *SE* = 2.998, *p* < 0.01) indicated that individuals with OCD reported lower confidence than controls even when statistically controlling for normative certainty, a consistent metacognitive bias in line with preceding results. Second, we found a significant interaction effect between CC and group (*β* = 3.152, SE = 1.363, *p* < 0.05). This indicates a stronger alignment between confidence and CC in participants with OCD. The interaction between GC and group did not reach significance in this joint model (*β* = 1.328, *SE* = 0.785, *p* = 0.097; cf. Figure 4 B). For completeness, in a GC-only model specification (GC, group, covariates; without CC), GC remained significant (*β*=3.510, *SE*=0.895, *p*<0.001) and the GC × group interaction was also significant (*β* = 2.609, *SE* = 1.266, *p* < 0.05). Thus, GC does relate to confidence and shows a group interaction when entered alone; however, when GC and CC were modeled together (variance inflation factor [VIF]<3), the GC × group interaction was attenuated.

Taken together, while individuals with OCD exhibited a general negative metacognitive bias (i.e., pervasively lowered confidence), their dynamic updating of confidence was paradoxically more optimal (i.e., closer to the Bayesian observer’s certainty) than in controls, countering a dominant hypothesis in the field that lowered confidence in OCD stems from globally impaired evidence integration.

## Discussion

In this study, we investigated the optimality of confidence judgments in individuals with OCD, a condition characterized by excessive doubt and chronic underconfidence^9,27,28,35,36.^ Employing a deterministic rule-shifting task combined with normative Bayesian modeling, our findings reveal a striking dissociation: despite lower confidence levels (metacognitive bias) relative to healthy controls, individuals with OCD exhibited superior trial-by-trial integration of performance feedback and evidence, demonstrating confidence updates that were more optimal as they were more closely aligned with a Bayes-optimal observer. Importantly, their objective performance was comparable to controls, indicating that reduced baseline confidence does not stem from impaired decision making but rather from a stable calibration bias. Our findings challenge notions of broadly impaired metacognition in OCD and instead propose the coexistence of two distinct metacognitive phenomena.

The first of these entails a chronic bias in baseline confidence calibration, manifesting as reduced subjective confidence irrespective of actual performance, available feedback, or Bayes-optimal certainty. This stable offset represents a classic metacognitive bias, a concept distinct from metacognitive sensitivity (i.e., how well confidence tracks accuracy moment-to-moment)^1,37,38^. It also resonates with extensive literature documenting pervasive, subjective doubt and lowered confidence in many individuals with OCD^24–30^, frequently uncoupled from actual task performance or objective outcomes^27,31,34,36,39^. Such a baseline calibration difference may reflect core alterations in internal evaluations of certainty^40^, independent of external validation or success.

Conversely, the second mechanism encompasses an enhanced capacity for integrating and dynamically responding to trial-specific evidence. Participants with OCD demonstrated significantly greater sensitivity to immediate feedback, updating their confidence judgments more accurately in relation to Bayesian-derived choice certainty compared to controls. This may be linked to hyperactive error-processing^27,41–43,43–45^ and increased reward prediction error signaling seen in OCD^46,47^. Rather than indicative of impaired processing, this heightened responsiveness may reflect an adaptive cognitive strategy, allowing accurate performance despite persistently low confidence. Indeed, this heightened evidence sensitivity echoes findings of superior performance in other contexts, like increased information sampling^4,8^.

These dual metacognitive profiles, impaired stable confidence calibration coexisting with enhanced dynamic evidence tracking, suggest differential involvement of neural substrates or cognitive systems. Baseline confidence alterations, manifesting as a negative metacognitive bias, might implicate hypoactivity in cortico-striatal circuits associated with internal belief states, goal-directed action, and reward valuation^48,49^. Conversely, enhanced dynamic evidence tracking, reflecting superior sensitivity to immediate feedback and errors, could be mediated by hyperactive fronto-cingulate systems involved in rapid performance monitoring, conflict detection, and exaggerated error signaling^42,43,46^. Both hypo-^50,51^ and hyper-activation^52,53^ and -connectivity in cortico-striatal and fronto-cingulate circuits in OCD may underpin the distinct metacognitive mechanisms identified here. Further investigation is required to elucidate how these different levels within the metacognitive hierarchy interact and influence one another^54–57^.

Our findings refine and contextualize previous research on metacognition and cognitive flexibility in OCD, using a deterministic task to dissociate confidence calibration from updating. Prior studies using probabilistic tasks have shown that individuals with OCD can track environmental contingencies and sometimes exhibit increased learning rates in action updates, without consistently showing reduced confidence relative to controls^34,58^. Others have reported reduced confidence and heightened responsiveness to feedback in behavioral adjustments^27^. In probabilistic environments, reductions in confidence may reflect adaptive responses to ambiguity and individual strategy profiles, complicating interpretation in the absence of a normative benchmark. By employing a deterministic paradigm with unambiguous feedback, we showed that individuals with OCD retained intact confidence updating relative to controls, despite globally reduced confidence.

This task structure may also explain why, consistent with some studies^12–14^ but not others^15–20^, participants with OCD in our study achieved accuracy comparable to controls, albeit with longer deliberation times. Removing time pressure and enforcing progression, unlike classical paradigms that often elicit apparent inflexibility^15–17,20^ likely permitted deliberate, compensatory strategies. The longer RTs in the OCD group are consistent with such adaptations. These findings reinforce that both cognitive and metacognitive processes in OCD (and beyond) may be sensitive to task structure and contextual demands. Moreover, we retained clinical realism in our cohort, with comorbidities present and a subset stably SSRI-treated. Although such heterogeneity can increase variance and attenuate some group contrasts, it strengthens external validity and ensures that the effects we report reflect OCD as it presents in practice. Future work should test whether the dissociation between baseline calibration and dynamic tracking generalizes across OCD subgroups, symptom dimensions, and learning environments with varying degrees of ambiguity and structure.

In conclusion, we show that metacognitive alterations in OCD do not represent a simple failure of judgment. Our findings suggest separable processes of consistently lowered confidence and superior evidence tracking. This reframes our understanding of the disorder, moving beyond a narrative of generalized deficits to one of specific, and potentially opposing, alterations in metacognition. Clinically, this may mean that pathological doubt arises not because patients cannot see the evidence, but because even when they see it more clearly than others, they cannot translate it into a stable feeling of certainty. This insight shifts the focus for future research and treatment towards belief calibration and enhancing subjective certainty independent of external validation and feedback integration.

## Methods

### Participants

We recruited 31 patients with OCD and 30 healthy controls (with no medical or psychiatric diagnoses) via the Yale OCD Research Clinic’s participant pool and social media platforms (Instagram, Facebook, and Craigslist). All participants were based in the United States, aged 18 to 55, and underwent group-specific screening procedures. To be eligible for the study, patients with OCD had to be unmedicated or stably treated with selective serotonin reuptake inhibitors (SSRIs). Diagnoses of participants with OCD who participated in this study were established by a licensed psychologist and/or psychiatrist using an unstructured clinical diagnostic interview and the Mini International Neuropsychiatric Interview (MINI)^59^. Within approximately two weeks of behavioral testing, the severity of their obsessions and compulsions was assessed using the Yale-Brown Obsessive Compulsive Scale (Y-BOCS)^60^. Patients had to have a minimum total score of 16 (moderate severity of symptoms) to be included in the study. Participants recruited as controls had never been diagnosed with any mental health disorder in the past. Controls underwent an additional clinical online assessment using the MINI and the Padua Inventory-Washington State University Revision (PI-WSUR)^61^, and were enrolled in the study as controls if they did not meet the criteria for any psychiatric diagnoses and did not indicated any obsessive-compulsive symptoms on a clinical level on the PI-WSUR (cf. Supplemental Information for more details on recruitment and clinical assessment).

The target sample size was determined by referencing comparable studies in the field^17,18^. The study was approved by the Yale University Human Investigation Committee (HIC #0803003626) and was managed using the Research Electronic Data Capture tools (REDCap)^62^ hosted at Yale University. Participants provided written informed consent via REDCap. The mobile app used for collecting task data was also approved by the University College London Research Ethics Committee.

### Procedure

After study enrollment, participants were sent a link (through REDCap) to complete demographic questions and a cognitive ability assessment (ICAR), the total score of which served as a proxy for IQ^63^. Once the questionnaires were completed, participants were instructed to download the app the study was hosted on (the Brain Explorer; https://brainexplorer.net/) to their touchscreen device and use a study-specific code to access the appropriate cognitive-behavioral task (cf. below). Participants played a newly developed rule-shifting task based on the principles of the ID/ED shift task, a widely-used cognitive flexibility paradigm (cf. below). Participants were compensated for their time with $20 per hour.

### The Rule-Shifting Task

The rule-shifting task presented participants with two or three “amoeba-like” stimuli (depending on the difficulty level; cf. below), each comprising multiple dimensions-such as color or shape-and corresponding features (e.g., if the dimension is shape, features might be round or star-shaped). Importantly, within any differing dimension, each displayed stimulus carried a unique feature value. Participants were instructed to identify a “target stimulus” by determining which dimension (e.g., color or shape) was currently relevant and, within that dimension, which feature (e.g., round vs. star-shaped) was rewarded (cf. Figure 1A).

On each trial, participants selected one stimulus and rated their confidence in that choice on a scale from 1 (“totally guessing”) to 100 (“totally certain”). The confidence slider began in a random position, and moving it prompted an anchor label (e.g., “pretty unsure,” “pretty certain”) that updated as the slider was adjusted. After confirming their confidence rating, participants received feedback: a coin (+1 coin) if they chose correctly or a cross (0 coin) if they erred. Coins accumulated in the top-right corner of the screen.

Unannounced rule changes occurred every 6 to 11 trials (irrespective of participant’s performance), requiring participants to detect and adapt to new contingencies (cf. Figure 1B). Two types of rule shifts were implemented:

1. Intra-dimensional (ID) shifts - The same dimension remained relevant, but its rewarded feature switched (e.g., shape stayed relevant, but “star” was replaced by “round”).
2. Extra-dimensional (ED) shifts - The previously rewarded dimension (e.g., shape) became irrelevant, and a new dimension (e.g., color) became rewarding.

The task featured three levels of increasing difficulty (cf. Figure 1C): at level 1, participants saw two stimuli and encountered simple ID/ED shifts across 60 trials (two blocks of 30); at level 2, additional dimensions were introduced while maintaining two stimuli, for a total of 70 trials (two blocks of 35); at level 3, a third stimulus was added, increasing the complexity of identifying the correct dimension and feature. This level also consisted of 70 trials (two blocks of 35), bringing the total to 200 trials across all levels.

#### Task exclusion criteria and final sample characteristics.

We applied task-specific exclusion criteria that were in line with criteria used in similar decision-making tasks with confidence ratings. Participants were excluded from data analysis if their overall task performance was at or below chance level (at level 1 and 2, the chance level was at 50%, and at level 3 the chance level was at 33%; excluded patients: *N*=1; excluded controls: *N*=1). Additionally, one participant with OCD was excluded because they changed their medication between recruitment and task completion. We ensured the use of the full confidence scale by checking that participants’ confidence ratings were not the same for more than 90% of the trials (this was not the case for any participant). We also examined reaction times (RTs) as potential indicators of attention drops. Specifically, we log-transformed RTs for each participant and task difficulty level and then, after excluding the first trial of each block, computed an outlier threshold. Following recommendations by^64^, any trial whose log-RT exceeded three standard deviations was removed from the final dataset (this accounted for 0.36% of total trials).

This resulted in a gender-balanced final sample of 29 patients with OCD (15 identified as female, 12 as male and two as non-binary; *M*_*age*_ = 31, *SD*_*age*_ =11.43) and 29 controls (14 identified as female and 15 as male; *M*_*age*_ = 32, *SD*_*age*_ = 10.99; cf. Supplemental Information for clinical characteristics of the final sample). After applying these exclusion criteria, the groups were still matched on average age (Wilcoxon rank-sum test: *z* = −0.327, *p* = 0.744) and IQ (total ICAR score; *z* = 0.715, *p* = 0.471).

### Statistical analyses

We used MATLAB 2023a (MathWorks) for initial data preprocessing. Data analysis was implemented using custom code in Python 3.8.8 with the packages: scikit-learn^65^, scipy^66^, numpy^67^, pandas^68^, statsmodels^69^, pymer4^70^, pingouin^71^, seaborn^72^, and matplotlib^73^.

#### The Bayesian observer.

To establish a normative benchmark for participants’ trial-by-trial confidence, we developed and implemented a Bayesian observer model. This computational model simulates an ideal learner that processes the same experimental information as each participant and computes an optimal, dynamically updated estimate of decision certainty. The model operates under the following key assumptions: (1) The relationship between features and outcomes is deterministic (i.e., a specific feature is consistently rewarded until a rule shift). (2) Shifts in the rewarded feature can occur, and these may be within the same dimension (intra-dimensional) or to a new dimension (extra-dimensional). (3) No explicit signals indicate a rule change; the observer must infer shifts solely from trial-by-trial feedback.

##### Bayesian updating.

At the beginning of each task level, the observer starts with no preference for any particular feature. Its belief about each of the *N* unique features across the displayed stimuli, $P_{0}\left(f_{k}\right)$, are initialized to a uniform probability: $P_{0}\left(f_{k}\right)=1/N$. On each subsequent trial, the observer updates its belief, the posterior probability $P_{t}\left(f_{k}\right)$, about each feature $f_{k}$ being the currently rewarding one. This update occurs after observing the participant’s choice and the resulting reward outcome $r_{t}$ (coded as 1 if rewarded, 0 if not), using Bayes’ theorem:

(1) $$P_{t}\left(f_{k}\right)=\frac{P\left(r_{t}|f_{k}\right)\;\times \;P_{t-1}\left(f_{k}\right)}{\sum_{j=1}^{N}P\left(r_{t}|f_{j}\right)\;\times \;P_{t-1}\left(f_{j}\right)}$$

where $P\left(r_{t}|f_{j}\right)$ is the likelihood of outcome $r_{t}$ given $f_{k}$ is the rewarding feature, and the sum in the denominator normalizes the posterior distribution across all N features.

Given the observer’s knowledge that sudden rule changes can occur, if contradicting evidence is encountered that would otherwise lead to a state where all current hypotheses are invalidated (mathematically, this corresponds to the denominator in Equation 1 becoming zero) with all feature beliefs $P_{t}\left(f_{k}\right)$ reaching 0, the beliefs for all N features are reset to $P_{t}\left(f_{k}\right)=1/N$. This allows the observer to “re-evaluate” all hypotheses from a neutral standpoint and adapt to the new contingency, assuming it is aware of the occurring rule changes.

##### Likelihood function.

The likelihood term $P\left(r_{t}|f_{k}\right)$ quantifies the support the observed feedback $r_{t}$ provides for the hypothesis that $f_{k}$ is the rewarded feature. On each trial, learning is restricted to dimensions that differ across the displayed stimuli. Within those dimensions, each stimulus carries a unique feature value. Let $n_{uf,t}^{c}$ be the set of unique features present in the chosen stimulus on trial t. Then:

(2) $$P\left(r_{t}|f_{k}\right)=\left\{\begin{array}{cc} 1/n_{uf,t}^{c}, & \text{if}\;r_{t}=1\;\text{and}\;f_{k}\;\text{is}\;\text{in}\;\text{the}\;\text{chosen}\;\text{stimulus,}\\ 0, & \text{if}\;r_{t}=0\;\text{and}\;f_{k}\;\text{is}\;\text{in}\;\text{the}\;\text{chosen}\;\text{stimulus,}\\ 1/n_{uf,t}^{u}, & \text{if}\;r_{t}=0\;\text{and}\;f_{k}\;\text{is}\;\text{in}\;\text{an}\;\text{unchosen}\;\text{stimulus,}\\ 0, & \text{if}\;r_{t}=1\;\text{and}\;f_{k}\;\text{is}\;\text{in}\;\text{an}\;\text{unchosen}\;\text{stimulus.} \end{array}\right.$$

##### Quantifying Uncertainty and Mapping to Confidence.

By applying the Bayesian observer model to each participant’s task run, we generated three trial-by-trial normative estimates. The model’s uncertainty about which feature is rewarded is quantified by the entropy $S_{t}$ of the posterior distribution $P_{t}\left(f_{k}\right)$:

(3) $$S_{t}=-\sum_{k=1}^{N}P_{t}\left(f_{k}\right){log}_{2}P_{t}\left(f_{k}\right)$$

To translate this entropy into an intuitive 0–100 confidence scale, *Bayes-optimal General Certainty (GC),* was calculated. *GC* reflects the proportion of uncertainty that has been reduced from the maximum possible entropy:

(4) $$GC_{t}=\frac{{log}_{2}N\;-\;{log}_{2}S_{t}}{{log}_{2}N}$$

In addition, we introduced a measure intended to mirror the conceptual basis of participants’ confidence ratings. Let $F_{u,t}^{c}$ denote the set of unique features present in the chosen stimulus on trial t. We defined the certainty in the chosen stimulus (*Bayes-optimal Choice Certainty*, CC) as the sum of the posterior probabilities associated with these features:

(5) $$CC_{t}=100\sum_{f_{k}\in F_{u,t}^{c}}P_{t}\left(f_{k}\right)$$

We scaled the GC and CC values so these optimal confidence estimates would align with the 0–100 confidence scale participants used. We then compared these Bayes-optimal estimates to the actual confidence ratings participants provided. This allowed us to quantify how well participant confidence aligned with an ideal Bayesian strategy and to pinpoint any systematic deviations, such as consistent under-confidence or inefficient evidence integration.

#### Behavioral analyses

To characterize the link between task variables (e.g., difficulty level, dimensional shift) and participants’ behavior and characteristics (e.g., patient versus non-patient) in a trial-by-trial hierarchical manner, we constructed regressions as mixed-effects models, using the Lmer function from the pymer4 package in Python^70^. All regression models investigating group differences were covaried for age, gender, and an approximation of IQ.

Accuracy in this rule-shifting task was defined as a binary variable capturing whether participants had chosen the correct (coded as 1) or an incorrect stimulus (coded as 0). We z-scored predictors in our regression models except if they were categorical (gender: 1= female, 2= male, 0= non-binary/Other; group: 1= patient with OCD and 0= control) prior to the analysis to allow comparability of regression coefficients. To account for both population-level and individual-level effects, we included both fixed and random effects in our mixed-effects model, performed standard convergence diagnostics, and used BIC to compare candidate fixed- and random-effects structures, selecting the most parsimonious specification (cf. Supplemental Information). Covariates such as demographics were included as fixed effects.

For the regression models predicting confidence on trial t, we shifted the independent task variables such as accuracy and dimensional by one trial (t – 1) as participants only received feedback after rating their confidence on a given trial. An example model in the *pymer4* syntax would be as follows:

$${\mathrm{Confidence}}_{t}∼{\;\mathrm{Accuracy}}_{t-1}+\;\mathrm{Gender}\;+\;\mathrm{Age}\;+\;\mathrm{IQ}\;+({\mathrm{Accuracy}}_{t-1}|\;\mathrm{Participant})+(1|\;\mathrm{Task}\;\mathrm{Level})$$

We fit models with a binary outcome variable (e.g., accuracy: 0= incorrect, 1= correct) and set the family argument to ‘binomial’, resulting in a logistic regression. We compared group means using independent-samples t-tests. If normality or homogeneity assumption was violated, non-parametric alternatives were used. All tests were two-tailed and p-values were adjusted for multiple comparisons using the Bonferroni method where applicable (denoted as p*). Before these analyses, we identified and removed outliers using the interquartile range (IQR) method, excluding any data points above Q3+1.5×IQR or below Q1-1.5×IQR represent the first and third quartiles, respectively, and the IQR is defined as Q3-Q1.

## Supplementary Material

Additional Information

Supplementary Information is available for this paper.

Supplementary Files

This is a list of supplementary files associated with this preprint. Click to download.

• LoosenEtAISupplementalInformationMetacognitiveParadoxOfOCD.pdf

## Data Availability

The code to the main analyses is accessible in a GitHub repository, at https://github.com/DevComPsy/The-Metacognitive-Paradox-of-OCD. Anonymized data will be freely accessible in a linked OSF repository (https://osf.io/c6tnx/) upon publication.
